# Chronological Impact of Earthquakes on Blood Pressure: A Literature Review and Retrospective Study of Hypertension in Haiti Before and After the 2010 Earthquake

**DOI:** 10.3389/fpubh.2020.600157

**Published:** 2021-01-15

**Authors:** Ayman R. Fath, Amro Aglan, Jeri Platt, Jordan R. Yaron, Kyle S. Varkoly, Roxana N. Beladi, Diane Gorgas, Jean Tom Jean, Pierre Dasni, Abdullah S. Eldaly, Michael Juby, Alexandra R. Lucas

**Affiliations:** ^1^Internal Medicine Department, Creighton University, Phoenix, AZ, United States; ^2^Department of Medicine, Beth Israel Deaconess Medical Center, Harvard Medical School, Boston, MA, United States; ^3^Glen Echo Presbyterian Church, Columbus, OH, United States; ^4^Center for Personalized Diagnostics, Biodesign Institute, Arizona State University, Tempe, AZ, United States; ^5^Kansas City University, Joplin, Kansas City, MO, United States; ^6^Department of Emergency Medicine and Office of Global Health, Ohio State University's Wexner Medical Center, Columbus, OH, United States; ^7^Jerusalem Baptist Church, Fort-Liberté, Haiti; ^8^Santiago Medical School, Santiago, Dominican Republic; ^9^Plastic and Reconstructive Surgery Department, Tanta University Hospitals, Tanta, Egypt; ^10^Midwestern University Medical School, Phoenix, AZ, United States; ^11^Internal Medicine Department, Creighton University Arizona Health Education Alliance, Phoenix, AZ, United States

**Keywords:** Haiti, 2010 earthquake, blood pressure, hypertension, natural disasters (NDs), environmental stress, cardiovascular events

## Abstract

**Objective:** We review prior studies on the incidence of hypertension (HTN) after earthquakes and present a retrospective analysis of HTN after the 2010 earthquake in Haiti.

**Methods:** Prior reports on HTN incidence were reviewed and a retrospective chart review for diagnosis of HTN in 4,308 patient charts was performed over a 7 year period (five clinics). A retrospective cohort study (RCS) was then performed on 11 patients with linear follow-up.

**Results:** The Literature review revealed a significant increase in acute and subacute HTN following earthquakes. However, the chronic effects of earthquakes varied. Our chart review uncovered no significant difference in diagnosed HTN in a Fort-Liberté clinic 128 kilometers (km) distant and 4 weeks post-event. A secondary linear RCS for 11 individuals, prior to and after the earthquake, also did not detect a significant change in HTN prevalence.

**Conclusion:** Prior studies demonstrate acute and subacute, increases in HTN after earthquakes, but late changes have varied. Retrospective studies in the Fort-Liberté clinic, 128 km distant and 4 weeks post-event, revealed no significant change in HTN, confirming prior findings that changes in HTN after earthquakes are early and local events. Further work examining HTN after earthquakes is needed to improve early health care after natural disasters.

## Introduction

### Environmental Stress and Physical Conditions

Populations are prone to various forms of stress including physical, psychological, and environmental stress. Environmental stress is defined as environmental changes, including pollution, climate change, and natural disasters that cause psychological and/or physical stress. Population-based stress associated with natural disasters is well-documented in the literature. Individuals respond to stress via internal physiologic mechanisms, called allostasis, that involve activation of the sympathetic nervous system and the hypothalamic-pituitary-adrenal (HPA) axis, resulting in a release of high levels of catecholamines and cortisol in the blood (1). These stressors are predicted to lead to elevated blood pressure (BP), hypertension (HTN), and associated cardiovascular events. The exposure to overwhelming stressors impairs the homeostasis of these mechanisms and ultimately leads to emotional, behavioral, and/or physical adverse outcomes and specifically risk for increased cardiovascular events with uncontrolled HTN. In some cases, prior studies have demonstrated a significant association with a stimulus-response relationship between exposure to natural disasters and physical conditions such as cardiovascular diseases (2, 3).

### Earthquakes and HTN

The pathophysiology of earthquake-related cardiovascular events is believed to be induced by activation of the sympathetic nervous system together with HTN, endothelial dysfunction, increased platelet activation, and vascular thrombosis. Endothelial dysfunction is closely associated with activation of the coagulation cascades and enhanced inflammation as well as vasospasm. High levels of inflammatory biomarkers such as C-reactive protein (CRP), interleukins, tumor necrosing factor alpha, (TNFα), von-Willebrand factor (vWF), hematocrit, and D-dimer concentrations have been reported following natural disasters and earthquakes supporting this hypothesized mechanism for stress-induced cardiovascular complications including hypertension (3–6).

### Earthquakes and Cardiovascular Events

Earthquakes, like most natural disasters, create cumulative community stress through the sheer magnitude of immediate loss of shelter, health, financial stability, and food security, but also have lasting impacts on communities for months or years. In prior reports, the onset of acute cardiovascular events occurring after earthquakes has been variable, with cardiovascular events occurring acutely on the day of the event or a few days after the disasters. Cardiovascular complications that have been reported include Takotsubo cardiomyopathy, coronary occlusion with myocardial infarction (MI), cerebrovascular occlusion with cerebrovascular accident (CVA, strokes), and sudden cardiac death. Subacute and chronic outcomes potentially include heart failure. Venous thrombosis with deep vein thrombosis, with or without subsequent pulmonary embolism is also reported (7). Sites in near proximity as well as early times post events have demonstrated greater changes in HTN, and cardiovascular events as might be predicted.

### Haiti and Natural Disasters

**Figure 1:**
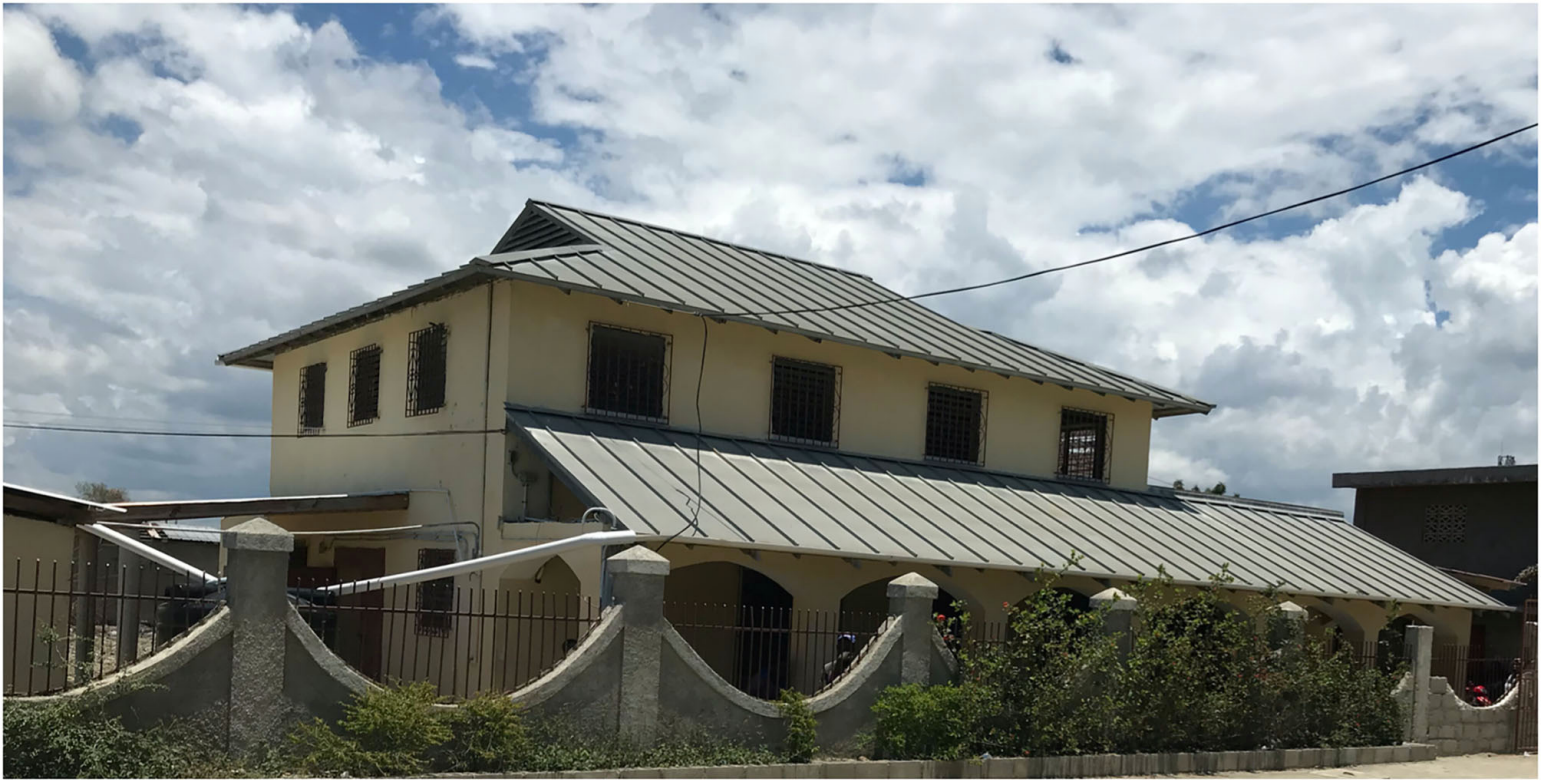
Fort-Liberté medical mission clinic.

Haiti has been impacted by a series of natural disasters throughout its history. These natural disasters are superimposed on extreme poverty and limited health care, placing Haiti in a vulnerable position among Low-Income Countries (LICs). The location of Haiti also makes it vulnerable to various forms of natural disasters that have contributed to Haiti being considered the poorest country in the Western Hemisphere. The Haitian population has been exposed to stress during the Torrential rains in 2004, Tropical Storm Fay, Hurricanes Hanna and Gustav in 2008, the October 2010 Cholera epidemic as well as a major earthquake in January 2010, and Hurricane Matthew in 2016. The 2010 Haiti earthquake that occurred on January 12th, 2010 is considered to be the worst natural disaster to impact Haiti in terms of loss of life, with ~300,000 deaths being attributed to this earthquake. The epicenter of this 2010 earthquake was near Port-au-Prince. More than a million people were displaced, contributing to a population increase of 75% in Fort-Liberté, along the northern coast of Haiti, within 1 month of the earthquake. For the past 15 years, we have made yearly medical service trips to Haiti and examined a diverse population of local residents through a small church-sponsored clinic in Fort-Liberté (Figure 1).

Reports of elevated BP and HTN have differed with variations in HTN incidence seen with proximity to the earthquake epicenter as well as the timing of HTN assessment after the event. Given prior variations in the reported incidence of HTN observed at later times after earthquakes and at sites remote from the epicenter, we have reviewed prior published literature examining the effects of earthquakes on the incidence of hypertension. We have also performed a retrospective analysis of the incidence of hypertension prior to and at 4–5 weeks or more after the 2010 earthquake in Fort-Liberté, Haiti, a site 128 km distant from the earthquake epicenter in Port-au-Prince. The review of prior literature is presented first providing the context for our retrospective analysis.

## Methods

### Literature Review of Prior Reports on Blood Pressure Changes and the Incidence of Hypertension After Earthquakes

Prior studies examining the effects of earthquakes on the incidence of HTN after earthquakes were identified. A literature search using the keywords of earthquake, BP, hypertension, and pregnancy was performed using PubMed, Google Scholar, Science Direct, UpToDate, and Research Gate searches to locate relevant articles. We identified 21 papers reporting changes in HTN incidence after earthquakes from China, Japan, the United States, and Italy from years 1995 to 2019.

### Retrospective Chart Review

An initial retrospective chart review was performed using data from an outpatient clinic affiliated with the Jerusalem Baptist Church in Fort-Liberté, Haiti for each of 5 years where data was available from the clinic charts; 2007, 2009, 2010 (4–5 weeks or more after the earthquake), 2011, and 2014, that included 4,308 total patients. The clinic was interrupted in years 2008, 2012, and 2013 due to hurricanes, Cholera, and political unrest. This retrospective chart review was approved by the clinic director and all patient data were de-identified prior to collating and analysis.

Data were collected in the local Fort-Liberté clinic charts and documented by volunteers. Basic demographic data and visit diagnoses were collected and reviewed, with a focus on a diagnosis of HTN. After review, patients younger than 18 years of age were excluded and a total of 2,876 patients were included in the analysis. BP was measured by a standard manual sphygmomanometer (BP cuff) with readings recorded during the clinic visits by either the visiting nurses or physicians on the medical team. Hypertension was defined as BP greater than systolic 130 mmHg and diastolic >90 mmHg. The prevalence of diagnosed HTN (BP ≥130/90 mmHg) was calculated and compared before and after the 2010 earthquake. Actual BP measurements were not provided in all of the charts.

To examine linear changes in individual patients who were seen consistently before and after the 2010 earthquake, a subset of 11 patients in this clinic was identified with consistent documentation of BP measurements over a period of 6 years from 2007 to 2012. Data from this smaller subset of patients was analyzed for changes in mean systolic blood pressure (SBP), diastolic blood pressure (DBP), and the number of BP medications and compared before and after the 2010 earthquake.

### Statistical Analysis

Data from the clinic documents were separated by date and included patients older than 18 years. The numbers and percentages of all patients with a diagnosis of HTN in the included cohort for each day and for the clinic charts for each year were calculated. Data from the initial retrospective chart review were analyzed by one-way Analysis of Variance (ANOVA) with a Fisher's LSD, Kolmogorov–Smirnov (KS) test, and Mann–Whitney using GraphPad Prism 8.4.3. For the linear cohort of 11 patients, a simple regression analysis was performed together with non-parametric Kolmogorov–Smirnov (KS) statistical test.

## Results

### Literature Review of Studies of Blood Pressure Change After Earthquakes

Blood pressure changes and hypertension incidence post-earthquake have been reported in multiple studies (Table 1) but have varied in the detection of HTN after earthquakes with varying acute, subacute, and chronic effects at sites near and remote from the epicenter. Variations in the numbers of people examined were also present. In Table 1, we reviewed prior reports on the association of earthquakes with changes in measured BP and the incidence of HTN recorded proximate to earthquake events in China, Japan, and Italy.

**Table 1:** Prior published studies examining blood pressure changes and hypertension incidence after earthquakes.

	**Patient Numbers (mean age)**	**Method of BP measurement**	**Timing of BP measurement pre-earthquake**	**Timing of BP measurement post -earthquake**	**Observed changes in BP (in mmHg): Mean ± SBP/±DBP**
**Hanshin-Awaji Earthquake (China−1995)**					
Kario et al. (8)	3	24-h; clinic	Morning before	9 weeks	Patient A: Clinic- +11/+3; 24 h- +22/+15; Daytime- +24/+17; Nighttime- +21/+13 Patient B: Clinic- +16/+5; 24 h- +26/+16; Daytime- +25/+15; Nighttime- +28/+18 Patient C: Clinic- +16/+3;24 h- +17/+17; Daytime- +16/+15; Nighttime- +18/+21
Kario et al. (9)	3	24- h; clinic	Morning before	Patient A: 9 months; Patient B: 11 months; Patient C: 8 months	Patient A: Clinic- +16/0; 24 h- +4/−1; Daytime- +4/+1; Nighttime- +0/+0 Patient B: Clinic- +19/+7; 24 h- −3/−1; Daytime- −5/−1; Nighttime- +1/+2 Patient C: Clinic- +1/+2; 24 h- +0/+6; Daytime- −4/+1; Nighttime- +11/+17
Kario et al. (4)	42 (68)	Clinic	Median BP of 3 consecutive visits from November 1, 1994- January 16, 1995	1-2 weeks	+18/8
Minami et al. (10)	36 (65): Group A- *n* = 16, < 50 km from epicenter; Group B- *n* = 20, > 50 km from epicenter	Home	1 week before	0–4 weeks	Group A: Week 0: +7/+2; Week 1: +2/+1; Week 2: +3/+2; Week 4: −1/+1 Group B: Week 0: +1/+1; Week 1: −1/-2; Week 2: +0/+0; Week 4: −1/−1
Saito et al. (5)	221 (59)	Clinic	< 3 months before	4 weeks	105 patients in severely damaged areas: +5.0/+3.9; 116 patients in surrounding areas: +3.4/+2.0
Kario et al. (11)	124 (69)	24- h; clinic	< 3 years before	1–2 weeks	+14 ± 16/+6 ± 10
**Central Italy Earthquake (Italy 1998)**					
Parati et al. (12)	1	24- h	Morning before	3–4 hs	+20/+37
**Mid-Niigata Earthquake (Japan 2004)**					
Kamoi et al. (13)	222 diabetics (67)	Home	Morning before	3 and 6 months	3 months: +3/+1; 6 months: +3/−1
**Sichuan Earthquake (China 2008)**					
Chen et al. (14)	11 (62)	24- h	Morning before	13.8 ± 6.3 min	+24.7/+25.6
**L'Aquila Earthquake Italy−2009**					
Petrazzi et al. (15)	2: Patient A (59); Patient B (44)	24- h	Morning before	Day after	Patient A: +14.7/+11.0; Patient B: +19.8/+7.4
Striuli et al. (16)	2 (same as Perazzi et al. subjects)	Clinic	Morning before	2 year follow up from above study	Patient A: −7.1/+0.5; Patient B: −2.4/−8.4
Giorgini et al. (17)	47 (52): 24 Unchanged therapy; 17 Increased therapy; 6 Reduced therapy	24- h	Mean 6.9 ± 4.5 months before	Mean 14.2 ± 5.1 months	Unchanged therapy group: Clinic- +6.2/+3.6; 24 h - +6.5/+4.1; Daytime- +6.1/+3.8; Nighttime- +5.0/+4.4 Increased therapy group: Clinic- −2.3/+0.3; 24 h - −0.3/+0.2; Daytime- −1.0/−0.5; Nighttime- +0.1/+0.7 Reduced therapy group: Clinic- −2.5/+2.5; 24 h: +0.5/−1.5; Daytime- +3.0/+1.5; Nighttime- −6.0/−4.5
**Great East Japan Earthquake (Japan-2011)**					
Azuma et al. (18)	4,035 (41.0)	Clinic	2004 annual checkup	2005 annual checkup	Men: Usual duty staff- −1.1/−0.4; Ordinary operational staff- +0.2/+1.2; Overworked operational staff-+1.5/+0.3 Women: Usual duty staff- −1.7/−0.5; Operational staff- +0.3/+0.8
Satoh et al. (19)	142(68)	Home	1 day before	Morning after; 2 weeks; 4 weeks	Next morning: +2.4/+0.9; 2 weeks later: +1.9/no DBP data given; 4 weeks later: −0.9/no DBP data given
Tanaka et al. (20)	132 with CKD (68.5)	Clinic	Within 1–2 months before	1–3 weeks	+4/+3
Watanabe et al. (21)	38 CKD; 39 control (66)	Home	At least twice weekly for 2 weeks	Morning after; 1 week	CKD patients: Next morning- +7.1 SBP, 1 week after- +3.8 SBP; Control patients: Next morning- +3.4 SBP, 1 week after- +3.6 SBP
Ito et al. (22)	110 (68): 73 Hypertensive; 68 normotensive	Clinic	8 weeks before	Within 2 weeks	Hypertensive patients: +4.6/+0.6; Normotensive patients: +1.0/+0.6
Konno et al. (23)	240 public employees; 1,776 general population	Clinic	2010	2011	Public employees: +11.3/+7.8; General population: 1.9/+1.1
Ohira et al. (24)	31,252 (40–74); Follow-up: 21,989	Clinic	2008 and 2010	2011–2013	Evacuees: Men- +5.8/+3.4; Women- +4.4/+2.8 Non-evacuees: Men- +4.6/+2.1; Women- +4.1/+1.7
Konno et al. (25)	225 public employees and 1232 individuals	Clinic	2010	2011; 2012	Public employees: 2011: +9.9/+5.3; 2012: +5.4/+3.2 General population: 2011- −3.0/+0.2; 2012- −0.1/+1.4
**Ya-An Earthquake (China 2013)**					
Li et al. (26)	52 (73)	Clinic	2 days before	Day of admission	+6.1 mean SBP only

*BP, blood pressure; CKD, chronic kidney disease; DBP, diastolic blood pressure; SBP, systolic blood pressure*.

#### Acute Impact of Earthquakes on Blood Pressure

In prior reports, earthquakes have had an impact on BP readings. Elevated BP has been detected within minutes to days after the event. A study performed in Great Sichuan, China in 2008 at the time of the Wenchuan earthquake demonstrated acute changes in BP after the earthquake. This study measured the BP at the time of the earthquake in 11 patients who were undergoing ambulatory BP monitoring (14). Mean BP increased rapidly from 126/72 mmHg to 151/98 mmHg with an average time of roughly 14 min following the initial onset of tremors in all patients regardless of age, sex, or history of prior confirmed or suspected hypertension. In this group of patients, the BP remained elevated for 6 h after the earthquake. The nocturnal decline in BP following the earthquake was <10% of the daytime BP before the quake for all patients in the study, reflecting a loss of normal BP variation in all cases. The pathophysiology behind this early transient elevation of BP is believed to be due to sudden activation of the sympathetic nervous system (13).

#### Subacute Impact of Earthquakes on Blood Pressure

Other studies have revealed an early but a more sustained elevation in BP following earthquakes. Kario et al. have studied BP changes before and 7–14 days after the Hanshin-Awaji earthquake in China (4, 11). In one study, increased BP levels of ~+18/+8 mmHg (measured change in systolic/diastolic BP) were detected in 42 patients 1–2 weeks after the earthquake when compared with BP levels measured before the event (4). Suzuki et al. in their study including 15 patients, found that the risk of myocardial infarction was increased by 3.5 times in survivors after the first 4 weeks post-earthquake, with an increased incidence in women with increased reported stress (27).

These effects were more prevalent during nighttime hours which was not expected based on the fact that the parasympathetic nervous system has increased activation at night (3). Minami et al. reported an even higher BP increase for patients (*n* = 36) assessed near the epicenter when compared to those assessed over 50 kilometers from the epicenter (10).

#### Chronic Impact of Earthquakes on Blood Pressure

The chronic impact of earthquakes on measured BP has been unclear. Most prior reports concluded that increases in BP after earthquakes were transient and returned to baseline levels measured prior to the earthquake within a few weeks.

Kario et al. reported a return of BP levels in the studied population (*n* = 124) to baseline after 4–6 months (11). Satoh et al. found an early increase in BP at 1–2 weeks, but this was much smaller during the 4 weeks follow-up in 142 patients (19). Giorgini et al. evaluated 24-h ambulatory BP monitoring before and after (mean ± S.D. 6.9 ± 4.5/ 14.2 ± 5.1 months, respectively) the 2009 L'Aquila earthquake in 47 hypertensive patients with unchanged therapy. Their analyses demonstrated a significant elevation of 24-h, daytime, and nighttime systolic and diastolic BP measured after the earthquake when compared with BP measured before the earthquake (17). Ohira et al. found an elevated BP in all patients 3 years later, with a greater elevation in those who were forced to evacuate from the earthquake when compared to those who were able to stay at home (24).

In contrast, Konno et al. reported no significant change in BP within 1 year of the Great East Japan earthquake in the general population of the Watari town but did find a significant difference in the measured BP in public employees (*n* = 240). This was postulated to be due to the increased workload and stress during the long-term disaster relief operations performed by these workers specifically (23). Minami reported increased BP levels close to the epicenter of the Hanshin-Awaji earthquake in China (*n* = 16), but much smaller increases at sites > 50 km from the epicenter (*n* = 20) (10).

Most studies investigating the influence of earthquakes on BP have established an increased incidence of hypertension at the time of the disaster and at sites near the earthquake center, however, the effects of earthquakes on populations at late follow-up times and sites remote from the epicenter have varied. There is also less information available for the impact of earthquakes on the prevalence of hypertension in a single site, select population across many years. One study observed a chronic elevation in BP only in public workers, but not in the general population (25). With our study, we assess the impact of the 2010 Haiti Earthquake on the prevalence of hypertension diagnosis from 2007 to 2014 at a clinic in Fort-Liberté, Haiti.

### Analysis of Hypertension Prevalence Before and After the 2010 Haiti Earthquake

**Figure 2:**
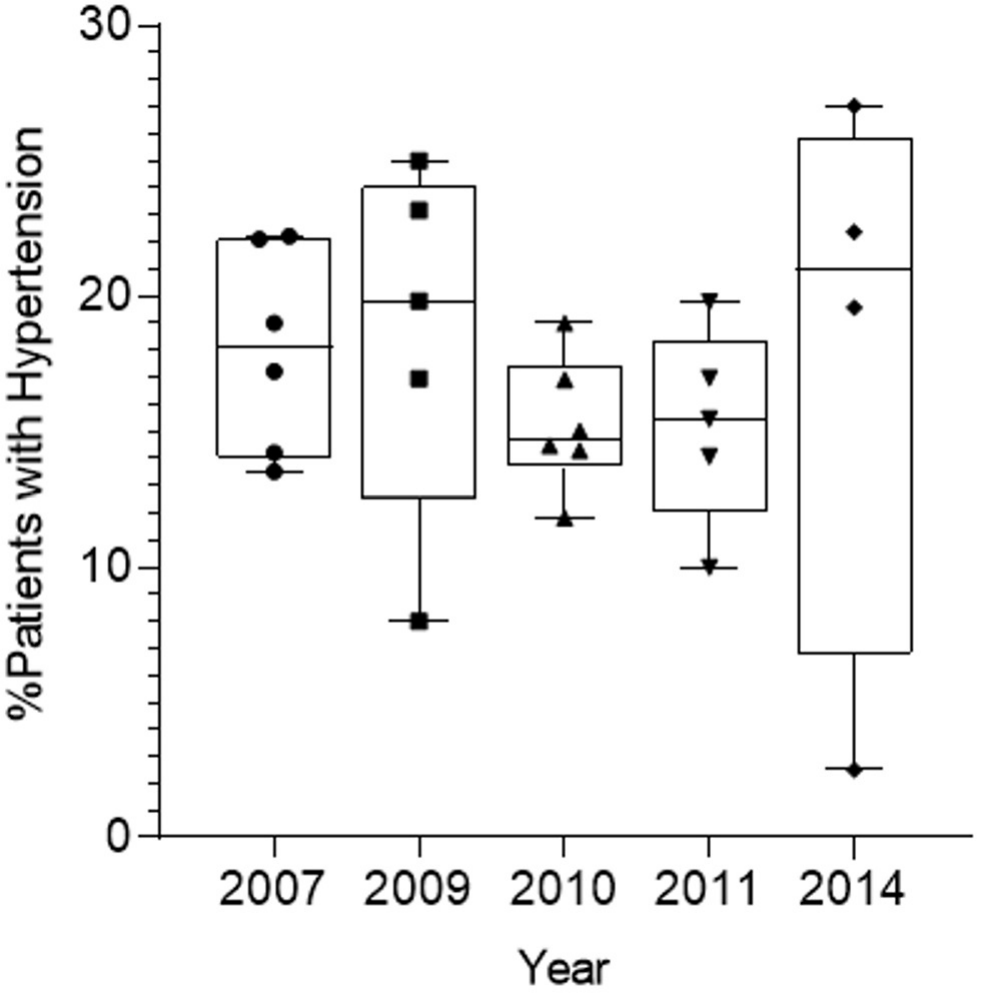
Prevelance of hypertension from 2007, 2009–2011, and 2014.

Hypertension was diagnosed as noted at 130/90 mmHg. The prevalence of diagnosed hypertension recorded in the clinic charts was 17, 20, 15, 16, and 19% in clinic years 2007, 2009, 2010, 2011, and 2014, respectively. The Fort-Liberté clinic was held 4–5 weeks after the earthquake. There was no statistically significant difference in the incidence of recorded HTN for each year, including for the year 2010 (Tables 2, 3; Figure 2).

**Table 2:** HTN prevalence 2007–2014.

	**2007**	**2009**	**2010**	**2011**	**2014**
Total	904	436	694	483	359
Age (mean ± SD)	Not reported	40.93 ± 18.07	40.13 ± 18.13	38.94 ± 19.28	41.66 ± 18.6
HTN	156	88	107	77	67
Prevalence %	17	20	15	16	19

*HTN, hypertension*.

**Table 3:** Statistical significance comparing various years.

**Uncorrelated fisher's LSD**	**95% CI for difference**	***P*-value**
2007 vs. 2009	−7.685 to 6.596	0.8755
2007 vs. 2010	−4.010 to 9.607	0.4023
2007 vs. 2011	−4.351 to 9.930	0.4257
2007 vs. 2014	−7.436 to 7.788	0.9621
2009 vs. 2010	−3.798 to 10.48	0.3413
2009 vs. 2011	−4.124 to 10.79	0.3631
2009 vs. 2014	−7.190 to 8.631	0.8516
2010 vs. 2011	−7.150 to 7.132	0.9979
2010 vs. 2014	−10.23 to 4.989	0.4816
2011 vs. 2014	−10.52 to 5.297	0.4996

**Figure 3:**
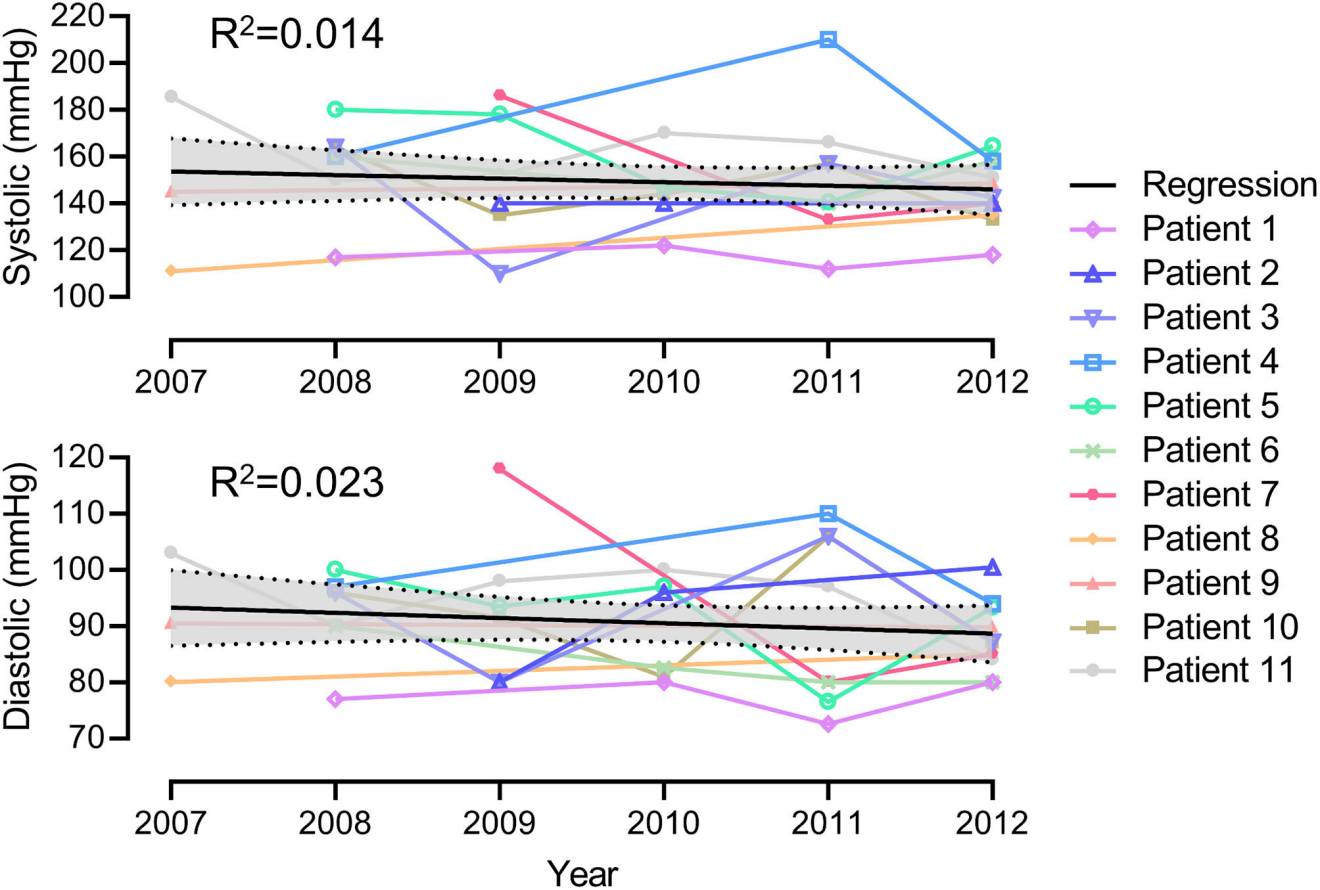
Linear regression analysis of mean blood pressure and the 2010 earthquake for the 11 patients.

**Figure 4:**
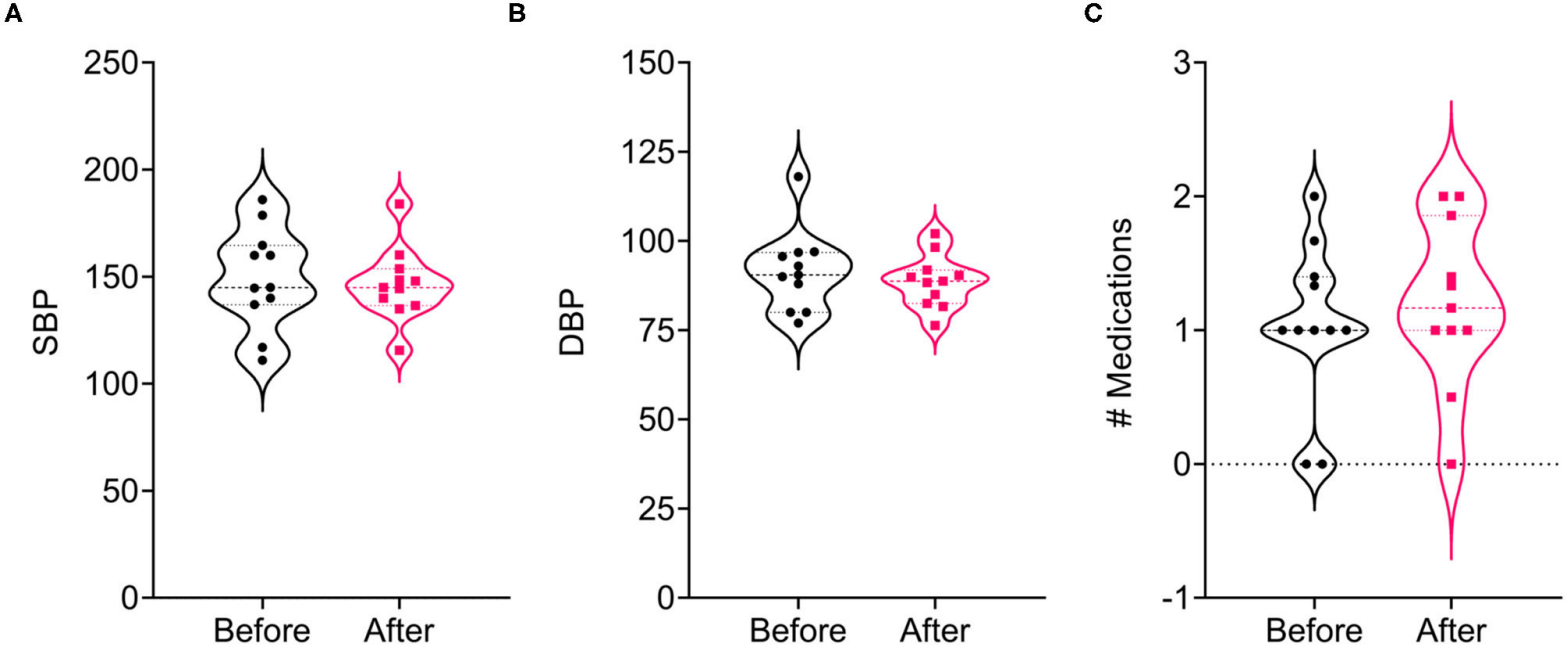
Kolmogorov–smirnov test on sbp **(A)**, dbp **(B)**, and medication change **(C)** for the 11-patient cohort before and after the 2010 earthquake.

In order to examine linear changes in individual patients seen in the clinic prior to and after the 2010 earthquake, mean SBP, DBP, and the number of BP medications for 11 individual patients were tracked and analyzed for a time span from prior to and after the 2010 earthquake; years 2007 and 2009 prior to the earthquake and years 2010, 2011, and 2014 after the earthquake. Blood pressure was not found to differ in this subset of patients (Table 4, *P* > 0.19). Linear regression of mean BP over the course of 6 years for these individual patients also revealed no association between the earthquake and altered BP (Figure 3, *R*^2^ < 0.02). Non-parametric Kolmogorov–Smirnov (KS) test analysis similarly did not reveal a statistically significant shift in mean SBP (Figure 4A), DBP (Figure 4B), or medications (Figure 4C) for these 11 patients over the 6 years assessed. The KS statistical analysis does not make the assumption that the two groups before and after the earthquake have the same standard deviations. With these serial BP measurements in the 11-patient cohort, we now have a timeline of changes in BP over a chronic reference time of analysis.

**Table 4:** Measured BP 3 years before and 2 years after the Haiti 2010 earthquake (for the cohort of 11 patients).

	**Before**	**After**	***P*-value**
Mean SBP ± SD (mmHg)	153.85 ± 24	145.74 ± 22.22	0.19
Mean DBP ± SD (mmHg)	92.8 ± 9.77	88.14 ± 11.5	0.08
Mean number of BP medications ± SD	1.19 ± 0.6	1.16 ± 0.71	0.85

*SBP, systolic blood pressure; DBP, diastolic blood pressure; SD, standard deviation; BP, blood pressure*.

In summary, our study examining BP and HTN at a clinic in Fort-Liberté, Haiti, 128 km distant from the earthquake epicenter at 4–5 weeks or more post-earthquake confirmed prior reports that demonstrated no change in HTN at remote sites and chronic times after these events.

## Discussion

This is the first study to investigate the prevalence of chronic hypertension before and after the 2010 earthquake in Haiti. This study includes a larger number of patients examined over multiple years in a small community in Northern Haiti before and after the earthquake and is compared to other studies on the prevalence of HTN after major earthquakes in other countries. We reviewed the published literature reporting changes in BP and HTN prior to and after major earthquakes. Most prior studies were performed over shorter follow-up times and incorporated a smaller number of patients. While most studies on BP and earthquakes point toward a positive correlation for HTN incidence at sites close to the epicenter and at early follow-up, these results have varied. With this study examining the incidence of HTN after the 2010 Haiti earthquake, we have also observed that there was no correlation between increased BP and earthquake prevalence at sites distant from the earthquake epicenter and at later times after the event. Even with the large influx of people from Port-au-Prince at the epicenter to the city of Fort-Liberté, there was no observed increase in HTN at this site and time post-event. There was also no detected statistically significant difference in the number of medications prescribed for HTN before and after the earthquake.

Our studied community was > 128 km from the epicenter of the earthquake and the patients were seen in the clinic 4–5 weeks after the earthquake. Additionally, communities may be built to handle environmental stressors differently and population density, socioeconomic status, religious devotion, and density of farmland may vary. Certainly, changes in measured BP and recorded HTN has varied with the individual prior studies as noted above.

The mechanism of sustained elevation of BP after earthquakes is hypothesized to be due to disruption of normal circadian rhythms as a result of psychological and physical stress from the earthquake. Two other contributory mechanisms have also been postulated: (1) activation of the HPA axis and its associated factors such as insulin resistance syndrome, accelerated atherosclerosis, and potential silent cardiovascular disease and (2) activation of the sympathetic nervous system responses. Both mechanisms have the potential to lead to endothelial dysfunction, increased blood viscosity, and platelet/hemostatic activation (Virchow's triad). Catastrophic stress is predicted to accelerate cardiovascular disease through hemodynamic changes such as HTN and thrombosis (1–3).

While we have examined a large number of charts, we should note that this study has some limitations; (1) the community studied is ~128 km from the epicenter and comparative analysis with Port-au-Prince was not available, (2) patient demographics were limited, (3) actual BP values were not consistently reported for all patients in the initial retrospective chart review thus a diagnosis of HTN was used for some charts, and (4) the data did not include all years from 2007 to 2014 due to interruptions in the clinic during major events such as political unrest (revolution), hurricanes, and epidemics; Data were available from years when the clinic was active. These recurrent instabilities in Haiti may have predisposed to mental and physiological resilience and make detection of changes in HTN incidence more difficult. It is important to note that while our acute care clinic is set up annually, presenting limitations on the true incidence of HTN in the total population, this data still presents accurate presentations of HTN in members of the community yielding partial data toward the true incidence of HTN of the encompassing population. Additionally, patient numbers do vary each year and our total patient number was higher in 2007, which may be due to many factors including attrition bias, voluntary response bias, or convenience.

One could suggest that our detection of the stability of BP after the earthquake may have been due to an influx of aid with improved care after the 2010 earthquake event, there was no significant increase in the numbers of HTN medications noted in the charts. Multiple traumatic stress-inducing events have occurred in Haiti including political unrest with the overthrow of the government, a cholera epidemic, hurricanes, and earthquakes. In fact, the cholera epidemic also occurred in October 2010, just 9 months after the earthquake. Thus, effects on HTN may be difficult to correlate with a single environmental disaster such as the earthquake in January 2010.

The reported prevalence of hypertension has varied in the Haitian population. Hypertension prevalence has been reported to be 43.6 and 42.3% in Léogâne (28). Another study measured hypertension prevalence to be 31.6% in Port-au-Prince, and 37.6% in Marmont (29). African American hypertension prevalence has been reported to be 42.4% for men and 44.0% for women in 2015 (30). Our lower incidence of hypertension in Fort-Liberté, Haiti, ranging from 15 to 20% in this cohort may provide another reason for the decreased prevalence seen in our study, both pre-and post-earthquake. In addition, the clinic is designed to provide acute care once every year to residents near and in Fort-Liberté, thus while this is a yearly, recurring clinic we may not see as many of the chronic HTN patients, but more often those with difficult to control HTN or other general medical or urgent problems.

### Clinical Importance

Hypertension is an important risk factor to assess after earthquake events. Long-standing risk factors for the development of coronary artery disease (CAD) have typically included age, serum cholesterol levels, high-density lipoprotein (HDL), BP and specifically HTN, and cigarette use, and also men at younger ages (31). A prior study of a cohort of 77,389 community-dwelling adults, aged ≥65 years, has examined a negative correlation between normotension and cardiovascular-related mortality, and a positive correlation between stage 2-3 hypertension and risk of cardiovascular-related mortality (32). This study posited the need to determine whether consistent BP control and more rigorous BP target ranges can improve early events and mortality after earthquakes. The dose-response relationship between the stage of hypertension and cardiovascular-related deaths has well-known associations; early adulthood HTN cases (mean 18.3 years), have an elevated risk of mortality from CVD, but not stroke (33).

Kario et al. reported an increase in the incidence of coronary heart disease-related mortality in elderly patients for a few months following an earthquake in Japan (34). Kabutoya et al. outlined the influence of the disruption of the hypothalamic-pituitary axis, endocrine system, and sympathetic nervous system and its effects in disrupting the chronic homeostatic stress response brought about by earthquakes (35). Aoki et al. identified the influence of both exogenous and endogenous factors such as drug discontinuation, increased salt intake, and an activated sympathetic nervous system in both the acute and chronic phases after an earthquake causing elevated BP (36). There remains a need to identify patients with chronic hypertension pre- and post-earthquake, or with acute elevations in BP after natural disasters such as earthquakes, to examine the incidence of cardiovascular-related mortality over a more extended time frame, such as in our smaller subset in our cohort study. Through the linear examination of this cohort of patients with hypertension pre-and post-earthquake in this study and examination of the chronicity of this hypertension, we can more accurately assess the role of environmental stress on cardiovascular-related mortality and treat these underserved communities more effectively.

## Conclusions

In conclusion, prior studies have examined both acute and subacute effects of earthquakes on BP elevation with variable correlations noted at sites removed from the earthquake epicenter and at later times after the event. Our retrospective study compares the impact of the 2010 Haiti earthquake on the prevalence of hypertension before and after the event and was the first to examine changes in BP in five different years over a 7-year period. There was no statistically significant difference in hypertension prevalence amongst patients from our clinic in Haiti from 2007, 2009, 2010, 2011, and 2014. Specifically, BP measured 4-5 weeks after the 2010 earthquake at a site 128 km (80 miles) north of the epicenter did not detect significant increases in BP, confirming prior work that has demonstrated less change in BP and less detected HTN at sites distant from the earthquake and at later follow up times. Furthermore, while many studies have demonstrated acute health impacts such as myocardial infarctions and strokes in victims of natural disasters, the chronicity of health issues such as HTN over several years in time may actually be preserved given the findings of this study.

We would suggest that in future studies a careful analysis of larger numbers of patients both early and late after natural disasters should be conducted to determine the risk of significant elevations of BP. Analyses of other risk factors, such as endothelial cell-derived von Willebrand's factor (vWF), C reactive protein (CRP), fibrinogen, renin, aldosterone, and troponin among other acute reactants might be measured. Von Willebrand's factor has been reported to remain elevated at 4-6 months post events, and it may be important to examine the risks of an earthquake-induced chronic hypercoagulable state secondary to prolonged elevation of risk factors (4). Finally, since hypertension is a well-known risk factor for cardiovascular-related mortality (28–30), it is important to assess the mortality of these patients with sustained hypertension post-earthquakes to further draw conclusions as to the impact of chronic hypertension on cardiovascular-related disease and mortality. By doing so, we can create specific treatment plans focused on BP management to improve long term as well as short term health in victims of environmental disasters.

## Data Availability Statement

The raw data supporting the conclusions of this article will be made available by authors upon request, without undue reservation.

## Author Contributions

ARL, JP, DG, JTJ, and PD collected data. ARF, AA, ARL, ASE, KSV, RNB, MJ, and JRY analyzed data. ARF, KSV, RNB, JRY, and ARL wrote the manuscript. All authors contributed to the article and approved the submitted version.

## Conflict of Interest

The authors declare that the research was conducted in the absence of any commercial or financial relationships that could be construed as a potential conflict of interest.
